# Interactions of SNPs in Folate Metabolism Related Genes on Prostate Cancer Aggressiveness in European Americans and African Americans

**DOI:** 10.3390/cancers15061699

**Published:** 2023-03-10

**Authors:** Hui-Yi Lin, Susan E. Steck, Indrani Sarkar, Elizabeth T. H. Fontham, Alan Diekman, Lora J. Rogers, Calvin T. Ratliff, Jeannette T. Bensen, James L. Mohler, L. Joseph Su

**Affiliations:** 1 Biostatistics Program, School of Public Health, Louisiana State University Health Sciences Center, New Orleans, LA 70112, USA; 2 Epidemiology and Biostatistics, and Cancer Prevention and Control Program, Arnold School of Public Health, University of South Carolina, Columbia, SC 29208, USA; 3 Department of Epidemiology, School of Public Health, Louisiana State University Health Sciences Center, New Orleans, LA 70112, USA; 4 Department of Biochemistry and Molecular Biology, College of Medicine, University of Arkansas for Medical Sciences, Little Rock, AR 72205, USA; 5 Winthrop P. Rockefeller Cancer Institute, Department of Epidemiology, Fay W. Boozman College of Public Health, University of Arkansas for Medical Sciences, Little Rock, AR 72205, USA; 6 Lineberger Comprehensive Cancer Center, University of North Carolina at Chapel Hill, Chapel Hill, NC 27514, USA; 7 Department of Epidemiology, Gillings School of Global Public Health, University of North Carolina at Chapel Hill, Chapel Hill, NC 27599, USA; 8 Department of Urology, Roswell Park Cancer Institute, Buffalo, NY 14203, USA; 9 Peter O’Donnell Jr. School of Public Health, University of Texas Southwestern Medical Center, Dallas, TX 75390, USA

**Keywords:** prostate cancer, aggressiveness, folate metabolism, genetic variants, SNP, interaction

## Abstract

**Simple Summary:**

Prostate cancer (PCa) is a complex disease. Identifying inherited genetic variants or single nucleotide polymorphisms (SNPs) for predicting PCa aggressiveness is essential for improving PCa clinical outcomes. However, the interactions of folate-related SNPs associated with PCa aggressiveness are understudied. The study’s objective is to evaluate interactions among the DHFR 19-bp polymorphism and 10 SNPs in folate metabolism and the one-carbon metabolism pathway associated with PCa aggressiveness. We evaluated 1294 PCa patients, including 690 European Americans (EAs) and 604 African Americans (AAs). None of the 11 individual polymorphisms were significant for EAs and AAs. For the EA PCa patients, the two SNP–SNP interaction pairs in *MTHFR-MTHFD1* and *MTHFR-SLC4A5* were significantly associated with aggressive PCa. For the AA PCa patients, the interaction of *DHFR*-19bp polymorphism and rs4652 (*LGALS3*) was significantly associated with aggressive PCa. These findings can provide valuable information for precision intervention and medicine of PCa aggressiveness.

**Abstract:**

Background: Studies showed that folate and related single nucleotide polymorphisms (SNPs) could predict prostate cancer (PCa) risk. However, little is known about the interactions of folate-related SNPs associated with PCa aggressiveness. The study’s objective is to evaluate SNP–SNP interactions among the DHFR 19-bp polymorphism and 10 SNPs in folate metabolism and the one-carbon metabolism pathway associated with PCa aggressiveness. Methods: We evaluated 1294 PCa patients, including 690 European Americans (EAs) and 604 African Americans (AAs). Both individual SNP effects and pairwise SNP–SNP interactions were analyzed. Results: None of the 11 individual polymorphisms were significant for EAs and AAs. Three SNP–SNP interaction pairs can predict PCa aggressiveness with a medium to large effect size. For the EA PCa patients, the interaction between rs1801133 (*MTHFR*) and rs2236225 (*MTHFD1*), and rs1801131 (*MTHFR*) and rs7587117 (*SLC4A5*) were significantly associated with aggressive PCa. For the AA PCa patients, the interaction of *DHFR*-19bp polymorphism and rs4652 (*LGALS3*) was significantly associated with aggressive PCa. Conclusions: These SNP–SNP interactions in the folate metabolism-related genes have a larger impact than SNP individual effects on tumor aggressiveness for EA and AA PCa patients. These findings can provide valuable information for potential biological mechanisms of PCa aggressiveness.

## 1. Introduction

Prostate cancer (PCa) is the most common incident cancer and the second leading cause of cancer death (11%) among American men [1]. PCa is a complex and heterogeneous disease. In the majority of cases, PCa is an indolent disease, although approximately 30% of PCa are aggressive with a high risk of progressing to lethal metastatic disease [2]. In addition, racial disparity in PCa incidence and mortality has been observed. African Americans (AAs) suffer a disproportionate burden of PCa, with 2.3 times higher PCa mortality rates and more aggressive PCa compared to European Americans (EAs) [3,4]. Therefore, identifying modifiable risk factors and genetic markers for aggressive PCa, particularly among AAs, who are at higher risk of virulent disease and have been underrepresented in research, is imperative for reducing the burden of this disease. Folate, a potentially modifiable factor for PCa, is a water-soluble B vitamin involved in DNA synthesis and repair and regulation of gene expression through DNA methylation as a methyl donor. The effect of folate on carcinogenesis is complex and depends on timing, dose, and type of cancer [5]. Several studies have shown that folate is significantly associated with PCa risk, but some did not verify this association [6,7,8,9,10,11,12,13,14,15,16,17]. A study showed that serum folate was positively associated with PCa risk, and PCa patients had a 10 nmol/L increase in serum folate compared with the controls in a population of African descent [18]. In addition, a recent meta-analysis based on seven studies showed that a high serum folate level was associated with increased PCa risk (odds ratio [OR] = 1.43) [19]. Another meta-analysis of six clinical trials found that PCa risk was significantly increased with folic acid supplementation (rate ratio [RR] = 1.24) [6]. In contrast, two other meta-analyses did not find an association between folic acid supplementation and PCa risk [20,21]. However, the number of studies evaluating folate and PCa aggressiveness is very limited.

The variability in unmetabolized serum folic acid is likely affected by genetic polymorphisms because it was not explained entirely by dietary intake [22]. It is well recognized that polymorphisms in folate pathway genes can modify folate levels and risk of cancers (such as colon cancer), such as dihydrofolate reductase (*DHFR*) and methylenetetrahydrofolate reductase (*MTHFR*) [23,24,25]. DHFR is the only enzyme involved in reducing folic acid and converting it into tetrahydrofolate [26]. The 19-bp deletion polymorphism in the *DHFR* gene could predict higher plasma concentrations of unmetabolized folic acid [27]. Previous studies have addressed the effect of *MTHFR* gene polymorphisms on PCa risk, but the results are inconsistent [28,29,30,31,32,33,34,35,36,37]. In addition, folate can affect one-carbon metabolism, which supports several physiological processes, including biosynthesis, amino acid homeostasis, epigenetic maintenance, and redox defense [38]. One-carbon metabolism genes have also been shown to impact DNA repair and gene methylation and are related to several cancers, including breast, colorectal, and liver [25,39,40]. The relationships between genes involved in one-carbon metabolism and PCa risk have also been investigated but less extensively, and these studies also produced conflicting results [11,30,41,42].

Identifying genetic markers for predicting PCa aggressiveness is imperative for improving PCa outcomes, especially for AAs at greater risk of high aggressive PCa. Most PCa genetic association studies have been conducted on men with European ancestry. The results from single nucleotide polymorphism (SNP) studies among EAs are challenging to apply to AA populations, where genomic variation may differ in types and frequencies. For example, the frequency of polymorphisms in *DHFR* and *MTHFR* genes differs by race, and associations between polymorphisms and circulating folate levels also vary by race [43,44,45,46,47,48]. In addition, some SNPs in one-carbon metabolism genes are significantly associated with high-grade PCa in white and black men, but these associations differ by race [49]. Furthermore, we are interested in two more genes: *LGALS3* and *SLC4A5*. Galectin-3 (*LGALS3,* also called *GAL3*) is commonly overexpressed by cancer cells and promotes cancer progression and metastasis for several cancers, such as PCa, breast cancer, and colon cancer [50]. *SLC4A5* is a member of the Na^+^ driven bicarbonate transporter (NDBT) family, whose expression levels are associated with hypoxia (low oxygen) [51]. The hypoxia tumor microenvironment has been shown to be associated with PCa aggressiveness [52]. The interactions of these SNPs associated with PCa aggressiveness are understudied. Therefore, the objective of this study was to evaluate whether interactions among the *DHFR* 19-bp deletion polymorphism and SNPs in genes in the folate metabolism pathway (*MTR*, *MTRR*, and *MTHFR*), one-carbon metabolism pathway (*MTHFD1, MTHFR, MTHFS*), two PCa-related genes (*SLC4A5* and *LGALS3*) can predict PCa aggressiveness in EAs and AAs.

## 2. Materials and Methods

### 2.1. Study Population

We included a total of 1294 PCa patients (690 EAs and 604 AAs) from the population-based North Carolina and Louisiana Prostate Cancer cohort (PCaP) [53]. In this study, the EA and AA groups are based on self-reported race. The PCaP cohort recruited men with the first diagnosis of histologically confirmed adenocarcinoma of the prostate who resided in the North Carolina and Louisiana study areas during 2004–2009. PCa patients were eligible to participate if they self-reported being EA or AA, were between 40 and 79 years old at diagnosis, could complete the study interview in English, did not live in an institution (nursing home), were not cognitively impaired, were not in a severely debilitated physical state, and were not under the influence of alcohol, severely medicated, or apparently psychotic at the time of interview. PCa aggressiveness is defined by a combination of Gleason score, clinical stage, and prostate-specific antigen (PSA) level at diagnosis as: (1) high aggressive (Gleason score ≥ 8 or PSA >20 ng/mL, or Gleason score ≥ 7 and clinical stage T3–T4); (2) low aggressive (Gleason score < 7 and stage T1–T2 and PSA < 10 ng/mL), and (3) intermediate aggressive PCa (all others). In order to reduce the potential misclassification of disease aggressiveness status, 1338 PCa patients (717 EAs and 621 AAs) with SNP data diagnosed with highly aggressive and low aggressive PCa were considered. Among them, 1294 patients who had genetic ancestry information were included in this study. The genetic ancestry proportions of European ancestry and African ancestry for each participant were estimated based on the fifty ancestry informative markers. The details of genetic ancestry were reported previously [54].

### 2.2. Genotyping

Genotyping of the *DHFR 19-bp* deletion (del) polymorphism, *MTR*, *MTRR*, *MTHFR*, *MTHFD1*, *MTHFR*, *MTHFS*, *SLC4A5*, and *LGALS3* was conducted at the Winthrop P. Rockefeller Cancer Institute at the University of Arkansas for Medical Sciences. The *DHFR* 19-bp deletion (del)/insertion (ins) polymorphism was analyzed with the TaqMan SNP genotyping assays (Thermo Fisher Scientific, Waltham, MA, USA) on the 7900HT Fast Real-Time PCR system (Thermo Fisher Scientific, Waltham, MA, USA). Primers and probe mix were available as premade and validated TaqMan genotyping assays, and all PCR reactions were carried out with the TaqMan Genotyping Master Mix. Briefly, reactions were heated to 95 °C for 10 min and subjected to 40 cycles of amplification at 95 °C for 10 s and 60 °C for 1 min. PCR amplification was followed by allelic discrimination plate reading and analysis. For quality control, blinded repeats of approximately 5% of samples were included. All SNPs had a call rate greater than 95%.

### 2.3. Statistical Analyses

Participants’ age and study site status by EAs and AAs were summarized using descriptive statistics. The age distribution by race was compared using the t-test, and the study site by race was tested using the chi-square test. All analyses were performed separately for EAs and AAs. The linkage disequilibrium (LD) status among the SNPs on the same chromosome was tested using r^2^. For each SNP, three different inheritance modes (additive, dominant, and recessive) based on the minor allele were evaluated. For testing SNP individual effects associated with PCa aggressiveness (high vs. low aggressiveness), logistic regression was applied. For each SNP, the best model with the smallest *p*-value among the three inheritance modes was selected. Additionally, we evaluated SNP–SNP interactions among the DHFR 19-bp polymorphism and 10 selected SNPs from the seven target genes associated with PCa aggressiveness. For SNP–SNP interaction analyses, we tested a total of 55 SNP/polymorphism pairs among the candidate polymorphisms. The SNP–SNP interaction analyses were performed using the logistic-model-based SNP interaction pattern identifier (SIPI) approach [55]. All models were adjusted for age, study site, and genetic ancestry.

**Figure 1:**
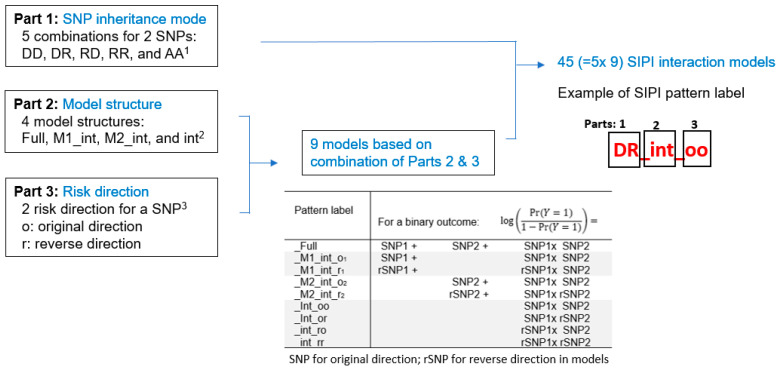
Summary of the 45 SNP–SNP interaction models based on the SNP Interaction Pattern Identifier (SIPI). Note: ^1^ D: dominant, R: recessive, A: additive inheritance mode. ^2^ Full: full interaction model with two SNP main effects plus an interaction; M1_int: main effect of 1st SNP plus an interaction; M2_int: main effect of 2nd SNP plus an interaction; and int: an interaction only. ^3^ Original direction is based on the minor allele, the reverse direction is based on the major allele. _o_1_, _r_1_: original direction, and reverse coding of 1st SNP (original coding for 2nd SNP). _o_2_, _r_2_: original direction, and reverse coding of 2nd SNP (original coding for 1st SNP). _oo, _or, _ro, _rr: 1st letter for 1st SNPs and 2nd letter for 2nd SNP.

SIPI tests 45 biologically meaningful interaction patterns for SNP–SNP interactions for each pair by considering three key features, which reflect the 3 parts of the SIPI model labels. As shown in Figure 1, the 1st part is the SNP’s inheritance modes (additive, dominant, and recessive), the 2nd part is model structure (hierarchical and non-hierarchical interaction models), and the 3rd part is risk direction (original and reverse). Based on two SNPs, there are 9 genotype combinations. The conventional approach for testing 2-way SNP–SNP interactions is the full or hierarchical interaction model with 2 SNPs with the additive inherited mode (coding as 0, 1, and 2) and their interaction. Using this full model to detect SNP–SNP interactions tends to lead to false negatives because it only tested one complicated interaction pattern. For importing detection accuracy, SIPI intensively searches 45 interaction patterns/models. As shown in Figure 1, there are 9 possible models by considering model structure and risk direction for each combination of inheritance mode. SIPI considers 4 model structures: the full interaction model (‘Full,’ both main effects plus interaction), the models with one main effect and interaction (M1_int or M2_int), models with only an interaction (such as int_oo), and 2 risk directions with the original (‘o’) direction based on the number of minor alleles and reverse (‘r’) direction. By integrating 5 combinations of inheritance modes (Part 1), there are a total of 45 (=5 × 9) SNP–SNP interaction patterns (such as DD_Full, DD_M1_int_o1, DD_M1_int_r1, DD_M2_int_o2, DD_M2_int_r2, DD_int_oo, DD_int_or, DD_int_ro, and DD_int_rr for the dominant-dominant mode). With this design, SIPI can combine genotype sub-groups with a similar risk profile or a small size for enhancing prediction power. The details are described previously [55]. For each SNP pair, the interaction pattern with the lowest Bayesian information criterion (BIC) value among the 45 testing patterns was selected. For multiple comparison justification, the Bonferroni correction criterion was *p*-value < 0.0045 (=0.05/11) for individual SNPs and *p*-value < 0.0009 (=0.05/55 pairs) for SNP–SNP interactions. However, it is well-known that Bonferroni correction is conservative. Thus, we applied the bootstrap internal validation method for the top SNP pairs with a *p* < 0.05 for selecting the promising pairs. The bootstrap method, a resampling technique, has been used in SNP association studies to reduce false positive findings [56]. In this bootstrapping, 500 samples are repeatedly drawn from the original data. In each bootstrap sample, a significant result was defined based on whether a SNP pair followed the 3pRule approach, which is the modified significance criterion by considering the *p*-values of its 2 SNPs’ individual effects (*p*-value of interaction pair (*p*-pair) < 0.01, *p*-pair< *p*-SNP1, and *p*-pair < *p*-SNP2). The percentage of the significance for each SNP pair based on 500 bootstrap samples was calculated. The significant SNP pairs were defined as a *p*-pair < 0.05 with a significance percentage greater than 65% out of the 500 bootstrap samples. The R SIPI package version 1.22, which can be accessed at https://github.com/LinHuiyi/SIPI, was applied to detect individual SNP effects and SNP–SNP interactions [55].

## 3. Results

For the 690 EA PCa patients, the mean age was 64.0 years (standard deviation [SD] = 7.7), and 53.8% were from the Louisiana site. For the 604 AA PCa patients, the mean age was 61.8 years (SD = 7.8), and 55.5% were from the Louisiana site. As shown in Table 1, 21.4% of EAs and 30.6% of AAs had high aggressive PCa, and the study site distribution was similar in both race groups (*p* = 0.541). There was high consistency between self-reported race and genetic ancestry status. The mean ancestry proportion of European ancestry was 96.7% (median = 98.8%) for self-reported EAs, and the mean ancestry proportion of African ancestry was 90.6% (median = 97.7%) for self-reported AAs. All of the target genes are protein-coding genes. The details of these genes are listed in Table 2. We tested linkage disequilibrium (LD) for SNPs on the same chromosome. There are 4 SNPs (rs2274976, rs1801131, rs1801133, and rs1805087) on chromosome 1 and 3 SNPs on chromosome 14 (rs4644, rs4652, and rs2236225). For EAs, only rs4644 and rs4652 were strong LD (r^2^ = 0.86), and others on the same chromosome had weak LD (r^2^ < 0.3). For AAs, all SNPs on the same chromosome had weak LD (r^2^ < 0.2).

Details regarding the *DHFR* 19-bp polymorphism and 10 selected SNPs from theseven genes for EAs and AAs are listed in Table 3. The minor alleles of the nine SNPs are consistent for EAs and AAs, except rs4652 in *LGALS3*. For rs4652, the ‘C’ allele was a minor allele (minor allele frequency [MAF] = 0.42) for EAs but was a major allele for AAs (‘C’ allele frequency = 0.84). Most minor allele frequencies for the 10 SNPs differed by race. Using rs1801133 in *MTHFR* as an example, the MAF of the ‘A’ allele was 33% for EAs and 13% for AAs. As shown in Table S1, all genotype distributions for the 10 SNPs and the del/ins status for *DHFR* 19-bp polymorphism were significantly different by race. For rs10380 in *MTRR*, the TT genotype was only 1.5% for EAs but was 11.3% for AAs (race difference, *p* = 1.7 × 10^−45^). For rs4652 in *LGALS3*, the CC genotype was 19.2% for EAs but 72.4% for AAs (race difference, *p* = 1.2 × 10^−87^). The *DHFR* 19-bp del/del genotype prevalence was 17.7% for EA and 31.0% for AA PCa patients (race difference, *p* = 2.5 × 10^−9^). The individual effects of the selected SNPs/polymorphism associated with PCa aggressiveness by considering three inheritance modes for EAs and AAs are shown in Table 3. Among the 11 polymorphisms, none of them were significantly associated with PCa aggressiveness for both EAs and AAs (all *p*-values > 0.05).

**Figure 2:**
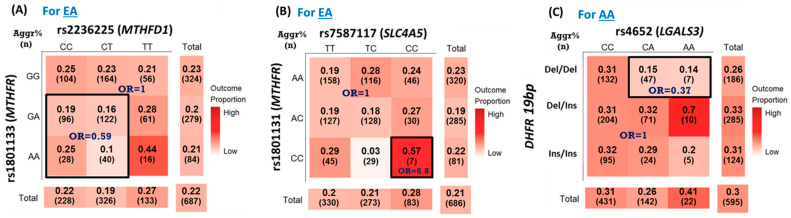
Three selected SNP–SNP interactions associated with prostate cancer aggressiveness. (**A**) rs1801133 (*MTHFR*)-rs2236225 (*MTHFD1*) in European Americans (EAs) with the DR_int_or interaction pattern, OR = 0.59 (95% confidence interval [CI] = 0.40–0.88), *p* = 0.009. (**B**) rs1801131 (*MTHFR*)—rs7587117 (*SLC4A5*) in EAs with the RR_int_oo interaction pattern, OR = 6.8 (95% CI = 1.39, 33.28), *p* = 0.018. (**C**) interaction of *DHFR*-19 bp polymorphism and rs4652 (*LGALS3*) in African Americans (AAs) with the DD_int_ro interaction pattern, OR = 0.37 (95% CI = 0.17–0.81), *p* = 0.012. Note: In each cell, the first value is the prevalence of prostate cancer aggressiveness, and the 2nd value in parenthesis is the sample size in each genotype combination.

The top SNP–SNP interaction pairs with a *p* < 0.05 associated with PCa aggressiveness for EAs and AAs are shown in Table 4 and Table 5, respectively. None of the SNP pairs in EAs and AAs reached the Bonferroni correction criterion (all *p* > 0.0009). Among them, three pairs (two pairs for EAs and one pair for AAs) were selected based on the bootstrap approach with >65% times of significance out of 500 bootstrap samples. For EAs, the SNP pairs rs1801133- rs2236225 in *MTHFR* and *MTHFD1 (p* = 0.009, 68.8% significance) and rs1801131- rs7587117 in *MTHFR* and *SLC4A5* (*p* = 0.018, 69% significance) were significantly associated with PCa aggressiveness (Table 4). The interaction between *MTHFR* rs1801133 and *MTHFD1* rs2236225 was significantly associated with PCa aggressiveness with a pattern of DR_int_or, an original-dominant and reverse–recessive interaction-only model (Figure 2A). This pattern indicated that EA PCa patients with the ‘GA/AA + CC/CT’ genotype in rs1801133 and rs2236225, respectively, suggested a lower risk of developing aggressive PCa (OR = 0.59, *p* = 0.009) compared to those with other genotypes in this SNP pair. As shown in Table 4 and Figure 2B, the interaction between rs1801131 *MTHFR* and rs7587117 in *SLC4A5* was associated with PCa aggressiveness (*p* = 0.018). The SIPI selected the RR_int_oo pattern, an interaction-only model with an original-recessive mode for both SNPs. As shown in Figure 2B, this RR_int_oo pattern indicated that the EA PCa patients with the CC+ CC genotype combination of rs1801131-rs7587117 had a higher risk of aggressive PCa compared to those with other genotypes in this SNP pair (OR = 6.8, *p* = 0.018). For the EA PCa patients, the top two high-risk groups of PCa aggressiveness were the CC+ CC genotype of rs1801131- rs7587117 (57%) and the AA+TT genotype of rs1801133- rs2236225 (44%), while the overall PCa aggressiveness prevalence was 21%.

For SNP–SNP interaction analyses for AAs (Table 5), there was one SNP pair significantly associated with PCa aggressiveness (*p*-value = 0.012, 65.4% bootstrap significance) out of the seven pairs with a *p* < 0.05. This SNP pair was the interaction of DHFR-19bp polymorphism and rs4652 in *LGALS3* with an interaction pattern of DD_int_ro, an interaction-only model with a reverse-dominant for DHFR-19bp polymorphism and original-dominant mode for rs4652. As shown in Figure 2C, AA PCa patients with the del/del status in the DHFR-19bp polymorphism and the rs4652 CA or AA genotypes had a lower risk of PCa aggressiveness (OR = 0.37, *p* = 0.012). The PCa aggressiveness prevalence for AA PCa patients with *DHFR*-19bp del/del and CA/AA in rs4652 was 14–15% compared with the overall PCa aggressiveness prevalence of 30%. For the AA PCa patients, the high-risk group of PCa aggressiveness was the del/ins+ AA genotype of *DHFR*-19bp-rs4652 (70%), while the overall PCa aggressiveness prevalence was 30%.

## 4. Discussion

We identified three SNP–SNP interaction pairs significantly associated with PCa aggressiveness: rs1801133 (*MTHFR*)-rs2236225 (*MTHFD1*) and rs1801131 (*MTHFR*)-rs7587117 (*SLC4A5)* for EAs and *DHFR*-19bp-rs4652 (*LGALS3*) for AAs. However, none of the individual effects of the DHFR 19-bp polymorphism and 10 target SNPs associated with PCa aggressiveness were significant. To our knowledge, the three SNP–SNP interaction pairs for PCa aggressiveness have not been reported. However, SNPs in some genes involved in these SNP pairs associated with PCa outcomes have been reported. The SNP of rs1801133 in *MTHFR* is related to PCa risk [57]. Another *MTHFR* SNP (rs9651118) has been reported to be associated with PCa recurrence with and without adjusting for known risk factors [58]. Another study with most Caucasians did not find associations between rs1801131 and rs1801133 in *MTHFR* and rs2236225 in *MTHFD1* with PCa risk, localized, and advanced PCa [30].

For interactions, SNP interactions between *MTHFR* and *MTHFD1* related to other clinical outcomes have been reported [59,60]. The SNPs between *MTHFR* and *MTHFD1* are associated with anterior encephalocele, a rare congenital anomaly of the central nervous system related to genetic defects in folate metabolism [59]. In this study, we also found SNPs in *LGALS3* and *SLC4A5* interacted with folate-related genes associated with PCa aggressiveness. *LGALS3* expression is associated with PCa progression and is a suggested PCa prognostic marker and therapeutic target [61]. In addition, galectin-3 is a proteolytic substrate for the serine protease PSA [62]. The non-synonymous SNPs rs4644 and rs4652 generate histidine-to-proline and threonine-to-proline polymorphisms in the galectin-3 protein at amino acids 64 and 98, respectively [63]. Proline introduces a Phi angle, creating a bend in the protein’s secondary structure [64]. These alterations in secondary structure may alter the function of the galectin-3 protein at the molecular level and contribute to increased PCa aggressiveness. *LGALS3*, expressed in human prostate intraepithelial neoplasia lesions and metastatic lymph nodes, is a crucial molecule and a potential therapeutic target in PCa progression and metastasis [65]. Furthermore, increased dairy consumption is associated with PCa progression [66,67]. The galectin-3 protein binds to galactose containing glycans. Thus, increased plasma galactose from the diet may impact the function of circulating galectin-3. In addition, studies showed that *LGALS3* expression [68,69] and *SLC4A5* expression [51] affected oxidative stress in animal experiments. Oxidative stress does appear to downregulate expression of the SCL4A5, which would be expected to disrupt pH regulation [70]. Moreover, the link between oxidative stress and folate deficiency in animal experiments also has been reported [71,72]. These support the potential biological links of our identified SNP–SNP interactions.

For racial differences, most of the top SNP–SNP interaction pairs associated with PCa for EAs and AAs were different (Table 4 and Table 5), and racial differences of all 10 SNPs in the folate-related genes and the DHFR 19-bp deletion polymorphism were significant. The EA and AA groups’ MAF status for the folate-related SNPs tested in our study is very similar to the results in the NCBI SNP database [73]. In our study, the MAF of the two SNPs (rs1801131 and rs1801133) in *MTHFR* were higher in EA than in AA PCa patients. Similar racial differences of these *MTHFR* SNPs between EAs and AAs were also observed in a large-scale study with both gender groups based on the US national survey [43]. For *DHFR* 19-bp deletion polymorphism, AA PCa patients had more del/del genotype in DHFR 19-bp deletion polymorphism than EA PCa patients (31.0% vs. 17.7%, *p* < 0.0001) in this study. It has been shown that the *DHFR* 19-bp deletion polymorphism is common with del/del genotype frequencies ranging from 10.5% to 48% in different populations [47].

## 5. Conclusions

In summary, we identified three novel interactions of SNPs or polymorphisms in the folate metabolism pathway, one-carbon metabolism pathway (*MTHFR*, *MTHFD1*, and *DHFR*), *SLC4A5*, and *LGALS3* associated with PCa aggressiveness, although the individual effects of these SNPs were not significant. To our knowledge, this paper is the first to assess SNP–SNP interactions in folate-related pathways associated with PCa aggressiveness. Our study demonstrated that SNP–SNP interaction findings could provide better prediction than individual SNP effects in the folate-related pathways. The strengths of this study are the inclusion of two race groups (EAs and AAs) and the application of a powerful statistical approach for SNP–SNP interaction analyses. The limitation is a lack of external validation due to a relatively small sample size, so the bootstrap internal validation approach was applied to reduce false positivity. Thus, future large-scale studies are warranted to verify these findings and elucidate the biological mechanism of the identified SNP–SNP interactions. The SNP-SNP interactions discovered in this study may lead to further understanding of the mechanistic pathways regarding interactions of these genes and the discovery of future therapeutic options for PCa.

## Figures and Tables

**Table 1:** Summary of study participants’ age and study site.

Factor	European Americans (N = 690) N (%)	African Americans (n = 604) N (%)	*p*-Value ^1^
Age (year)			
Mean ± standard deviation	64.0 ± 7.7	61.8 ± 7.8	<0.001
Study site			
Louisiana	371 (53.8)	335 (55.5)	0.541
North Carolina	319 (46.2)	269 (44.5)	
Genetic ancestry %			
Mean ± standard deviation	96.7% ± 7.3%	90.6% ± 15.5%	-
Prostate cancer aggressiveness			
No	542 (78.6)	419 (69.4)	<0.001
Yes	148 (21.4)	185 (30.6)	

^1^ Based on *t*-test for age and chi-square test for study site and prostate cancer aggressiveness.

**Table 2:** List and information of the target genes.

Gene Symbol ^1^	Gene Full Name (Location)	Functional Annotation ^2^
*DHFR*	dihydrofolate reductase (5q14.1)	metabolic process, cellular process, multicellular organismal process, developmental process, single-organism process, response to stimulus, biological regulation, cellular component organization or biogenesis
*MTR*	5-methyltetrahydrofolate-homocysteine methyltransferase (1q43)	metabolic process, cellular process, multicellular organismal process, developmental process, single-organism process, response to stimulus, cellular component organization or biogenesis
*MTRR*	5-methyltetrahydrofolate-homocysteine methyltransferase reductase (5p15.31)	metabolic process, cellular process, single-organism process, biological regulation
*MTHFR*	methylenetetrahydrofolate reductase (1p36.22)	metabolic process, cellular process, multicellular organismal process, developmental process, single-organism process, response to stimulus, biological regulation, cellular component organization or biogenesis
*MTHFD1*	methylenetetrahydrofolate dehydrogenase, cyclohydrolase, and formyltetrahydrofolate synthetase 1 (14q23.3)	immune system process, metabolic process, cellular process, multicellular organismal process, developmental process, single-organism process, biological regulation
*MTHFS*	methenyltetrahydrofolate synthetase (15q25.1)	metabolic process, cellular process, single-organism process
*SLC4A5*	solute carrier family 4 member 5 (2p13.1)	metabolic process, cellular process, multicellular organismal process, developmental process, single-organism process, localization, biological regulation, cellular component organization or biogenesis
*LGALS3*	galectin 3 (14q22.3)	cell killing, immune system process, metabolic process, cellular process, biological adhesion, signaling, developmental process, locomotion, single-organism process, response to stimulus, localization, multi-organism process, biological regulation, cellular component organization or biogenesis

^1^ All are protein coding genes. ^2^ Extracted from the Goterm_Biological Process (BP)_1 using the DAVID Bioinformatics Resources (https://david.ncifcrf.gov/) accessed on 3 March 2023.

**Table 3:** Individual SNPs associated with prostate cancer aggressiveness by race.

				European Americans (n = 690)				African Americans (n = 604)			
SNP /Polymorphism ^4^	Chr	Position (GRCh38)	Gene	Min < Maj (MAF) ^1^	Mode	OR (95% CI) ^2^	*p*	Min < Maj (MAF) ^1^	Mode	OR (95% CI) ^2^	*p*
rs2274976	1	11790870	*MTHFR*	A < G (0.05)	Dom	0.88 (0.46–1.69)	0.702	A < G (0.03)	Dom	0.79 (0.37, 1.65)	0.525
rs1801131	1	11794419	*MTHFR*	C < A (0.33)	Dom	0.89 (0.61, 1.29)	0.532	C < A (0.17)	Rec	0.83 (0.26, 2.64)	0.746
rs1801133	1	11796321	*MTHFR*	A < G (0.33)	Dom	0.79 (0.55, 1.15)	0.223	A < G (0.13)	Add	0.72 (0.49, 1.07)	0.104
rs1805087	1	236885200	*MTR*	G < A (0.2)	Rec	1.33 (0.57, 3.14)	0.510	G < A (0.29)	Add	0.91 (0.69, 1.19)	0.495
rs7587117	2	74221528	*SLC4A5*	C < T (0.32)	Add	1.24 (0.95, 1.62)	0.112	C < T (0.16)	Rec	1.51 (0.57, 3.98)	0.408
rs10380	5	7897078	*MTRR*	T < C (0.1)	Dom	1.12 (0.7, 1.78)	0.645	T < C (0.34)	Rec	1.49 (0.88, 2.53)	0.139
rs4644	14	55138217	*LGALS3*	A < C (0.39)	Add	1.06 (0.81, 1.38)	0.683	A < C (0.26)	Rec	0.53 (0.24, 1.18)	0.120
rs4652 ^5^	14	55138318	*LGALS3*	C < A (0.42)	Dom	1.09 (0.74, 1.62)	0.661	A < C (0.16)	Rec	1.78 (0.71, 4.42)	0.217
rs2236225	14	64442127	*MTHFD1*	T < C (0.43)	Rec	1.46 (0.94, 2.28)	0.096	T < C (0.22)	Rec	1.84 (0.85, 3.99)	0.123
rs622506	15	79846853	*MTHFS*	C < A (0.35)	Rec	0.81 (0.43, 1.54)	0.529	C < A (0.18)	Rec	1.63 (0.75, 3.55)	0.219
DHFR-19bp ^3^	19		*DHFR*	Del < Ins (0.43)	Dom	0.88 (0.59, 1.31)	0.531	Ins < Del (0.45)	Dom	1.30 (0.88, 1.91)	0.189

^1^ Min < Maj: Minor allele < major allele; MAF: minor allele frequency (percentage). ^2^ Odds ratio (95% confidence interval), logistic models adjusted for age, study site, and ancestry. ^3^ Del: deletion; Ins: insertion. ^4^ All SNPs within the same chromosome had weak LD (r^2^ < 0.3), except rs4644 and rs4652 for European Americans had strong LD (r^2^ = 0.86). ^5^ Major and minor alleles reverse for EAs and AAs.

**Table 4:** SNP–SNP interactions associated with prostate cancer aggressiveness in European Americans.

SNP Pair	Gene1	Gene2	Pattern	OR (95% CI) ^1^	*p*-Value ^1^	Significance% ^2^
rs1801133_rs2236225	*MTHFR*	*MTHFD1*	DR_int_or	0.59 (0.40, 0.88)	0.009	68.8
rs1801133_rs4644	*MTHFR*	*LGALS3*	RD_int_or	0.22 (0.06, 0.73)	0.013	55.2
rs2236225_rs7587117	*MTHFD1*	*SLC4A5*	RR_int_rr	0.61 (0.41, 0.91)	0.014	58.2
rs1801133_ rs4652	*MTHFR*	*LGALS3*	RD_int_or	0.23 (0.07, 0.77)	0.018	52.2
DHFR-19bp_rs1805087	*DHFR*	*MTR*	RR_int_oo	6.26 (1.36, 28.76)	0.018	40.8
rs1801131_ rs7587117	*MTHFR*	*SLC4A5*	RR_int_oo	6.80 (1.39, 33.28)	0.018	69.0
rs1801133_rs1805087	*MTHFR*	*MTR*	DD_int_or	0.64 (0.43, 0.97)	0.034	50.8
rs1805087_rs2236225	*MTR*	*MTHFD1*	DR_int_oo	1.92 (1.04, 3.56)	0.038	44.8

^1^ Odds ratio (95% confidence interval) and p-value based on logistic models adjusted for age, study site, and ancestry for the European population. ^2^ The percentage of significance, which was defined as *p*-interaction < 0.01 and *p*-interaction< *p*-value of any of the two composite SNPs, based on the bootstrap validation with 500 runs.

**Table 5:** SNP–SNP interactions associated with prostate cancer aggressiveness in African Americans.

SNP Pair	Gene1	Gene2	Pattern	OR (95% CI) ^1^	*p*-Value ^1^	Significance% ^2^
DHFR-19bp_rs4644	*DHFR*	*LGALS3*	DD_int_rr	0.49 (0.29, 0.85)	0.011	52.2
rs1805087_rs2236225	*MTR*	*MTHFD1*	DR_int_ro	4.02 (1.38, 11.68)	0.011	57.4
DHFR-19bp_rs4652	*DHFR*	*LGALS3*	DD_int_ro	0.37 (0.17, 0.81)	0.012	65.4
rs1801131_rs4652	*MTHFR*	*LGALS3*	DR_int_ro	2.86 (1.07, 7.67)	0.037	36.4
rs10380_rs1805087	*MTRR*	*MTR*	RD_int_oo	1.98 (1.02, 3.83)	0.043	42.6
DHFR-19bp_rs10380	*DHFR*	*MTRR*	RR_int_ro	1.79 (1.02, 3.14)	0.043	40.6
rs10380_rs2236225	*MTRR*	*MTHFD1*	RD_int_oo	2.43 (1.02, 5.79)	0.045	43.4

^1^ Odds ratio (95% confidence interval), logistic models adjusted for age, study site, and ancestry for the African population. ^2^ The percentage of significance, which was defined as *p*-interaction < 0.01 and *p*-interaction< *p*-value of any of the two composite SNPs, based on the bootstrap validation with 500 runs.

## Data Availability

The data generated in this study are available upon request from the corresponding author and the North Carolina—Louisiana Prostate Cancer Project Management Committee https://pcap.bioinf.unc.edu/snapshot.php, accessed on 7 February 2023.

## Supplementary Materials

The following supporting information can be downloaded at: https://www.mdpi.com/article/10.3390/cancers15061699/s1, Table S1: Genotype distributions by race.
