# Supplementary material for: Interactions of SNPs in Folate Metabolism Related Genes on Prostate Cancer Aggressiveness in European Americans and African Americans
Source: Cancers (Basel). 2023 Mar 10;15(6):1699. doi: 10.3390/cancers15061699 (PMC10046243; doi:10.3390/cancers15061699)
Supplement: Supplementary file 1 [file cancers-15-01699-s001.zip › cancers-2237748-supplementary.pdf]

Table S1. Genotype distributions by race

	Allele	EA (n=690) N (%)			AA (n=604) N (%)			
SNP	A/B	AA	AB	BB	AA	AB	BB	p-value[a]
rs2274976	A/G	0 (0)	66 (9.6)	621 (90.4)	1 (0.2)	38 (6.3)	564 (93.5)	0.027
rs1801131	C/A	81 (11.8)	286 (41.6)	320 (46.6)	15 (2.5)	168 (27.8)	421 (69.7)	$3.8x10^{-19}$
rs1801133	A/G	84 (12.2)	281 (40.8)	324 (47.0)	10 (1.7)	134 (22.2)	460 (76.2)	$1.0x10^{-28}$
rs1805087	G/A	30 (4.4)	217 (31.5)	441 (64.1)	52 (8.6)	243 (40.3)	308 (51.1)	$2.9x\ 10^{-6}$
rs7587117	C/T	83 (12.1)	274 (39.9)	330 (48.0)	18 (3.0)	163 (27.0)	423 (70.0)	$2.5x10^{-17}$
rs10380	T/C	10 (1.5)	124 (18.0)	554 (80.5)	68 (11.3)	277 (46.1)	256 (42.6)	$1.7x10^{-45}$
rs4644	A/C	115 (16.7)	313 (45.6)	259 (37.7)	41 (6.8)	235 (39.2)	324 (54.0)	$4.2x10^{-11}$
rs4652	C/A	131 (19.2)	314 (46)	238 (34.9)	431 (72.4)	142 (23.9)	22 (3.7)	$1.2x10^{-87}$
rs2236225	T/C	133 (19.4)	326 (47.5)	228 (33.2)	29 (4.8)	209 (34.6)	366 (60.6)	$1.1x10^{-26}$
rs622506	C/A	78 (11.4)	324 (47.2)	285 (41.5)	28 (4.7)	161 (26.7)	413 (68.6)	$1.0x10^{-21}$
DHFR-19bp3	Del/Ins	122 (17.7)	344 (49.9)	223 (32.4)	187 (31.0)	291 (48.2)	126 (20.9)	$2.5\ x10^{-9}$

[a]Based on Fisher's exact or chi-square test for comparing genotype distribution vs. race
